# Platelet-rich plasma with versus without hyaluronic acid for hip osteoarthritis: a systematic review and meta-analysis

**DOI:** 10.3389/fbioe.2025.1545431

**Published:** 2025-03-24

**Authors:** Michael Silveira Santiago, Felipe Meireles Doria, José Morais Sirqueira Neto, Fabio França Fontes, Erick Sobral Porto, Felipe J. Aidar, Marcus Vinicius Vieira de Matos Pereira Silva, Deivyd Vieira Silva Cavalcante, Fatemeh Akbarpoor, Fernanda Valeriano Zamora, Davi Teixeira de Souza, Reuthemann Esequias Teixeira Tenorio Albuquerque Madruga, Alfonso López Díaz-de-Durana, María Merino-Fernandez, Rosana Cipolotti

**Affiliations:** ^1^ Health Sciences Graduate Program, Federal University of Sergipe, Aracaju, Brazil; ^2^ Department of Medicine, Tiradentes University, Aracaju, Brazil; ^3^ Department of Medicine, Federal University of Sergipe, São Cristóvão, Brazil; ^4^ Ribeirao Preto Medical School University of Sao Paulo, Ribeirao Preto, Brazil; ^5^ Department of Physical Education, Federal University of Sergipe, São Cristóvão, Brazil; ^6^ Department of Biology, Federal University of Maranhao, São Luís, Brazil; ^7^ Mohammed Bin Rashid University of Medicine and Health Sciences, Dubai, United Arab Emirates; ^8^ Department of Medicine, Rio de Janeiro State University, Rio de Janeiro, Brazil; ^9^ Department of Medicine, Federal University of Sergipe, Aracaju, Brazil; ^10^ Sports Department, Faculty of Physical Activity and Sports Science—INEF, Universidad Politécnica de Madrid, Madrid, Spain; ^11^ Facultad de Ciencias de la Salud, Universidad Francisco de Vitoria, Madrid, Spain

**Keywords:** hip, osteoarthritis, platelet, PRP, hyaluronic acid, viscosupplementation

## Abstract

**Background:**

The use of intra-articular orthobiologics in hip osteoarthritis (HOA) has been presented as a therapeutic option and to postpone arthroplasty. There is little scientific evidence on the clinical application of platelet-rich plasma (PRP) associated with hyaluronic acid as dual therapy. Thus, the aim of our systematic review is to compare the clinical improvement with the use of PRP with versus without hyaluronic acid (HA) in hip osteoarthritis.

**Methods:**

We systematically searched Cochrane, PubMed, and Embase databases for studies evaluating patients with HOA who received PRP with vs. without HA. Pain and functional score were collected and pooled at 3-, 6-, and 12-months follow-up. Mean differences (MD) and 95% intervals were calculated, and heterogeneity was assessed using I^2^ statistics. All statistical analysis was performed using R with the *meta* package.

**Results:**

We included 2 randomized controlled trials (RCTs) and 1 cohort study, comprising 190 patients, of whom 88 received the PRP plus HA. Relative to PRP alone, dual therapy led to significantly higher pain scores at 3 months (SMD 0.35; 95% CI 0.06 to 0.64; p < 0.01; I^2^ = 0%) and at 12 months (MD 11.92 points; 95% CI 3.87 to 19.97; p < 0.01; I^2^ = 0%), translating into worsening of pain including HA. There was no difference between groups at any follow-up regarding functional score or pain at 6 months.

**Conclusion:**

Joint infiltration in HOA with PRP combined to HA showed higher perception of pain scores. Our findings suggest that the addition of HA in PRP treatment does not bring significant improvement and worsens patients’ quality of life. However, more randomized trials with larger populations may increase robustness.

**Systematic Review Registration:**

identifier, CRD42024581335

## 1 Introduction

Mobility issues directly impact the quality of life. Cartilage integrity plays a fundamental role in the movement. Established osteoarthritis is characterized by disrupted joint homeostasis, the development of osteophytes, subchondral bone sclerosis, and a decrease in joint space, as observed on radiographic assessment. Clinically, the painful symptoms and functional impairment are disabling. The hip is the second joint most affected by osteoarthritis, with femoro-acetabular impingement, previous traumatic events, osteonecrosis of the femoral head and rheumatic disease being the main predisposing pathological conditions. In this context, chondral damage is increased with mechanical overload in high-impact activities or with the aging process. Hip osteoarthritis (HOA) is a common condition affecting the joint, surrounding structures, leading to pain, stiffness, and often impairment of daily living (Sambe et al., 2023).

Approximately 10%–25% of people over 60 years of age are affected by HOA, with a slightly higher incidence in men, placing a worrying disease by the economic burden of expensive treatment. When there is resistance to clinical therapy, non-operative procedures have grown in importance in recent years, mainly because they do not imply a worsening of chondral damage and provide another option for controlling joint pain (Kravos and Jerman, 2012). Orthobiologics are most commonly used in knee osteoarthritis with promising results; they are increasingly important in HOA, particularly for delaying or postponing hip replacement surgery for younger patients or when patients refuse more aggressive procedures, such as hip replacement (Zhao et al., 2020).

Inside this complex degenerative disease, the balance between cartilage production and its destruction gained focus in the recent scientific literature. The intervention strategy involves approaching the early stages, slowing down the progression of cartilage damage and total hip arthroplasty for advanced cases. Non-surgical treatments, such as cell therapy using platelet-rich plasma (PRP)extracted from peripheral blood, have gained popularity primarily for the repair of tendon and ligament injuries. Due to the benefits generated at the extra-articular level, clinical articular application has been extended, delaying the need for joint replacement (Lim et al., 2023). PRP is theorized to aid cartilage repair by stimulating cell activity, reducing inflammation to relieve pain, and improving joint function. It may also enhance joint lubrication by increasing synovial fluid viscosity, potentially slowing disease progression. However, more robust clinical research is needed to confirm its effects on cartilage health. There are different PRP formulations available depending on the leukocyte concentration and centrifugation process of obtaining, which implies a greater catabolic effect of cytokines to the detriment of the repairing anabolic action on platelets (Lim et al., 2023).

PRP is a blood-derived product rich in growth factors, vascular and transforming factors, in addition to a high level of platelets. During the process of obtaining the final PRP product, there are two or more centrifugation phases with different speeds, which bring about satisfactory clinical results. However, the lack of a specific protocol, depending on the number and speed of centrifugation, results in uncertain and poorly reproducible results (Tjandra et al., 2024). With the increasing use of PRP for osteoarthritis, there is still no optimal formulation of PRP with platelet counts and attention has been focused on leukocyte concentrations in PRP. In the study by Filardo et al., side effects were observed in the treatment of osteoarthritis compared to pure PRP (P-PRP), which had a lower concentration of leukocytes, possibly due to the release of pro-inflammatory cytokines by leukocytes. Therefore, the best clinical response was found with the P-PRP formulation (Xu et al., 2017; Filardo et al., 2012). Their use can be added to HA to stimulate chondrogenesis and inhibit degenerative enzymes, in addition to its mechanical properties (Ye et al., 2018). Although there is insufficient scientific evidence to support the widespread application of PRP in osteoarthritis, and its stimulation of chondrogenesis is not well-established *in vivo* or *in vitro*, it has shown potential as a promising therapeutic alternative, particularly for improving pain control (Owaidah, 2024).

The synergistic action of these biological products in joint applications has been further investigated in knee osteoarthritis, with promising results. For HOA, results in agreement with knee osteoarthritis are expected when combining orthobiologics as we have HA with higher molecular weight (MW) concentrations and better standardization of the use of PRP (Gazendam et al., 2021). Depending on the MW of HA, there are different clinical responses and varying effects on osteoarthritis, with pain relief, improved function, and postponement of surgery. Considering the safety profile, viscosupplementation may be an effective option in the treatment of chondral damage, with emphasis on the use of HA with higher MW (Lu et al., 2023). Clinical improvement in short-term outcomes appears to be influenced by the cellular composition of intra-articular PRP and the severity of osteoarthritis. The interaction between these factors warrants further investigation by the scientific community (Filardo and Kon, 2016).

With an understanding of the biochemical actions of HA for joint health in promoting improved viscosity, adhesion capacity and anti-inflammatory and analgesic effects, it is considered a biological product for use in joint diseases, playing a crucial role in maintaining joint stability and facilitating movement. Once osteoarthritis is diagnosed, we notice a 33%–50% decrease in the concentration levels of this natural orthobiological product and an increase in infiltrations, which disrupts the homeostasis of the joint environment. With the ease of access to ultrasound-guided procedures for hip infiltrations, we have seen greater use of HA, but the number of studies evaluating improvements in pain and joint function is still limited. Regardless of the joint being treated by HA, the physiological environment of an osteoarthritic joint improves by restoring protective viscoelasticity, reducing friction, and enhancing mobility (Leite and Buehler, 2018). In the search for an addition of anabolic effects with a reduction in inflammatory cytokines, dual therapy has been used, combining two orthobiologics.

The combination of PRP and HA in osteoarthritis was initially studied in the knee joint following promising results in laboratory animal models. This combination exhibits a synergistic biochemical action through independent mechanisms, facilitating cell signaling by releasing inflammatory molecules, catabolic enzymes, cytokines, and growth factors. The repair of degenerated cartilage is mediated by orthobiologics, which modulate the role of inflammatory cytokines in chondrocyte destruction through specific mediators such as CD44 and TGF-β. These actions theoretically inhibit the inflammatory response and slow the progression of osteoarthritis (Leite and Buehler, 2018; Chen et al., 2014; Andia and Abate, 2014). However, this association has been minimally investigated in hip osteoarthritis as it requires the use of ultrasound to guide the procedure (Zahir et al., 2021).

To avoid heterogeneity of the sample, recent systematic reviews have focused on comparing PRP plus HA versus PRP alone, particularly on knee degeneration, showing promising results when some outcomes were evaluated (Ivander and Anggono, 2024; Baria et al., 2022). The present meta-analysis aims to evaluate the influence of HA in combination with PRP compared to PRP alone in the therapeutic management of HOA in different follow-ups, considering pain and functional score outcomes.

## 2 Materials and methods

This systematic review and pairwise meta-analysis were conducted in accordance with the Cochrane Collaboration recommendations and Preferred Reporting Items for Systematic Reviews and Meta-Analyses guidelines and Protocols checklist (available in the online version of this article (Belk et al., 2022; Sterne et al., 2016). As such, its protocol was prospectively registered in PROSPERO database under the protocol CRD42024581335.

### 2.1 Eligibility criteria

In the study selection process, we started with deduplication and independently screened all potentially eligible studies. Four independent reviewers (FJA; FVZ; FMD; MCVF) and one validator (MSS) collaborated in combining outcomes from three databases and evaluated the studies for inclusion. Initially, they were excluded based on title and abstract if they did not pertain to the subject of interest. The remaining studies were then reviewed in full to verify eligibility. According to the inclusion criteria, two PRP centrifugation processes were accepted and we did not discriminate the leukocyte content of the final product to be infiltrated into the joint. We excluded trials not concluded, technical reports, editor responses, narrative reviews, systematic reviews, meta-analyses, non-comparative research, scientific posters, study protocols, conference abstracts not published as well as any pre-clinical studies or those not published in English. Unrelated papers not addressing the application of orthobiologics to treat hip osteoarthritis were also excluded.

We finished with a full independent reading of the papers by two authors, and we emphasized the presence of the following points: 1) peer-reviewed articles; 2) compared PRP in association with HA to PRP alone. Studies were excluded if they (1) were ongoing trials, not concluded; (2) were basic science research; (3) did not provide data on HOA patients; or (4) were case reports. We made no exclusions related to the publication date. Data extraction was manually performed.

Discrepancies were resolved through consensus between the reviewers and a third author (FJA) made the final decision in the event divergence was reached.

### 2.2 Search strategy

A comprehensive search was conducted across MEDLINE (via PUBMED), EMBASE (via OVID), and Cochrane databases from their inception until August 2024. No language restrictions were applied, and our search terms were a combination of Medical Subject Headings terms and keywords relating to “hip,” “osteoarthritis,” “platelet-rich plasma,” and “hyaluronic acid.” References from eligible studies and systematic reviews were also examined for additional relevant research. The specific search strategies for each database are available in Supplementary Table S1.

### 2.3 Clinical analysis tools

The data were extracted from eligible studies: study details—title, authors, publication year, study design, study definition, and inclusion/exclusion criteria; participant information—sample size, mean age, sex ratio, and HOA severity according to the Kellgren-Lawrence radiographic classification (K-L scale), which ranges from grade I (doubtful), grade II (mild), grade III (moderate), and grade IV (severe).

When extracting clinical data from articles, we choose outcomes compatible with *PICOTT* (population - HOA, intervention - Dual therapy, outcomes, control group- PRP alone, type of study-therapeutic, no restriction time) as main outcomes: pain, functional impairment. We used the numerical rating scale (NRS) ranging from 0 to 10 (or 100) mm as the data is reported, and functional questionnaires applied for general osteoarthritis, as the Western Ontario and McMaster Universities Osteoarthritis Index (WOMAC) score (from 0 to 100, in which 0 denotes the lowest degree of OA and 100 denotes the highest degree of OA) and in the hip joint, it was considered HARRIS HIP SCORE (HHS), ranging <70 is a poor result, 70–80 is fair, 80–90 is good, and a score of 90–100 is excellent. The final score of the functional questionnaires represented the current clinical condition. As the clinical improvement based on the final score in each questionnaire is with the inversion of the values upwards (increasing - HHS) and downwards (WOMAC), the tabulation was changed to a negative sign in the spreadsheet for the correct interpretation of the final data in the analysis in the statistical program. After treatment with each orthobiologic, final scores from these questionnaires were recorded.

### 2.4 Group analysis

Data extracted from studies included: 1) study details such as authors, sample size, intervention groups, and follow-up duration; 2) patient information including mean age, standard deviation, gender, and K-L scale (Kohn et al., 2016); 3) outcomes for pain and functional at 3, 6, and 12- months.

Due to the restricted number of studies at the end of the selection process and the availability of data, we choose a minimum of 2 studies for analysis according to the follow-up. Pain and functional scores outcomes were evaluated at 3-, 6- and 12- month follow-ups. In the 3-month pain outcome, all articles were included because they made *PICOOT* data available. In the functional score outcome, we only restricted the analysis to 2 articles in the 6-month follow-up due to lack of data.

In the intervention group of studies, we restricted the analysis to those who received the combination of PRP and hyaluronic acid. While in the control group, only in Palco et al. did the participants in the control group receive leukocyte-poor PRP (Palco et al., 2021).

### 2.5 Quality of evidence

In line with Cochrane guidelines, we employed the revised Cochrane risk-of-bias tool for non-randomized studies (ROBINS-I) to assess observational studies. The Cochrane risk-of-bias tool for randomized trials (ROB-2) was used to evaluate RCTs (Moher et al., 2009; Sterne et al., 2019). Pain was the primary outcome assessed using both ROBINS-I and ROB-2. Disagreements were resolved through consensus after discussion between the authors (MS and FV).

Considering that the level of evidence represents confidence in the information provided, the GRADE (Grading of Recommendation, Assessment, Development, and Evaluation) system was used, which assesses the quality of evidence for each available outcome. GRADE was a tool applied to assess the certainty of evidence in this Systematic Review, with four levels of classification: high, moderate, low and very low. Study design, publication bias, effect magnitude, dose-response gradient, and residual confounding factors were the main factors considered to determine the level of evidence.

### 2.6 Assessment of publication bias

Two review authors (FVZ; DVSC) assessed the risk of bias in each study by using the revised Cochrane risk of bias (RoB2/ROBINS-I tools). The examination domains included biases arising from the randomization process, deviations from intended interventions, missing outcome data, measurement of the outcome, and selection of the reported result. After responding to the signaling questions, one of three types of bias judgments was selected, namely “low,” “high,” and “some concerns.” In the case of conflicts, a third author (DTS) was contacted as an unbiased arbitrator. The limited number of studies (fewer than ten) precluded a quantitative analysis for small study effects or publication bias (Sterne et al., 2016).

### 2.7 Statistical analysis

Mean differences (MD) with 95% confidence intervals (CIs) were used as the measure of association when units were consistent; otherwise, standardized mean differences (SMD) were calculated when the measurements were reported in a non-identical way in each study, either with regard to the pain scale or the functional questionnaire (WOMAC/HHS) with individual interpretation for each final score. A restricted maximum likelihood (REML) random-effects model was applied using the inverse variance method (Schwarzer et al., 2019). As there were 03 studies in the systematic review, we limited the meta-analysis by comparing the means at different times, without using other statistical studies.

Statistical analyses were conducted with R software (version 4.3.0; R Foundation for Statistical Computing, Vienna, Austria). Heterogeneity was assessed using Higgins and Thompson’s I^2^ statistics. P-values less than 0.05 were considered statistically significant. Heterogeneity was categorized as high (I^2^ ≥ 50%), moderate (I^2^ 25%–50%), or low (I^2^ < 25%) (Deeks et al., 2019).

## 3 Results

### 3.1 Study selection and characteristics

**FIGURE 1:**
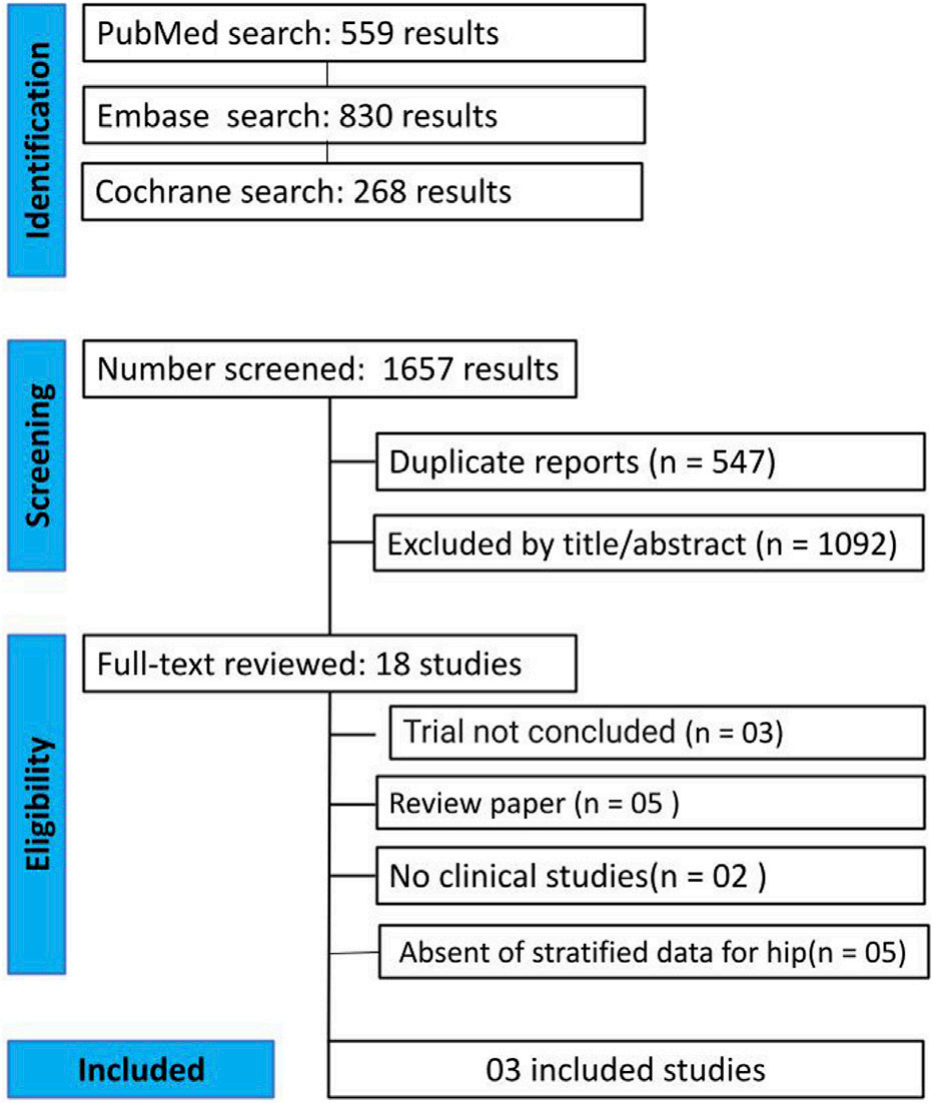
PRISMA (Preferred Reporting Items for Systematic Reviews and Meta-Analyses) Flow diagram of study screening and selection to the studies identified with the search strategy.

As shown in Figure 1, the initial search yielded 1,657 results. After a meticulous review of titles, abstracts, and full texts, we eliminated 547 duplicate entries, and 1,092 studies that did not meet the criteria were excluded. At final selection, 18 studies remained for evaluation based on predetermined inclusion standards. From these, 3 studies were selected: 2 RCTs and 1 cohort study. These studies involved 190 participants, with 88 individuals receiving dual therapy.

There was a similar distribution by gender in both the control group (PRP treatment alone or leukocyte-poor PRP) and the intervention group (dual therapy), with a mean age of 50.62–64.9 years, considering the lack of data available in Dallari et al. (Dallari et al., 2016; Nouri et al., 2022). Patients were included based on both the clinical criteria and a radiological grading system, using the K-L scale. 2 studies included patients with K-L grades 2 and 3, while the last study split the sample to all grades. The molecular weight of HA was reported in 2 studies and ranged from 1,500 to 3,200 kDa. The studies’ characteristics are presented in Table 1.

**TABLE 1:** Baseline characteristics of the included studies.

Author and year	Study design	K-L1(n)	K-L2(n)	K-L3 (n)	K-L4(n)	Hips	Follow-up (months)	Molecular weight (HA)kDa	Group	Sample size	Age (years)	Female
Dallari et al. (2016)	RCT	22	18	24	16	80	12	1,500–3,200	PRP + HA	31	MD	19
									PRP	44		24
Nouri et al. (2022)	RCT	0	33	30	0	63	6	2,500–3,200	PRP + HA	31	60.29	23
									PRP	32	58.22	22
Palco et al. (2021)	Cohort	0	24	28	0	52	12	MD	PRP + HA	26	64.81	14
									PRP	26	50.62	10

Abbreviations: RCT, Randomized Clinical Trial; K-L, Kellgren-Lawrence scale; PRP, Platelet Rich Plasma; HA, Hyaluronic Acid; MD, Missing Data.

### 3.2 Pooled analysis

#### 3.2.1 Pain at 3, 6, and 12 months

**FIGURE 2:**
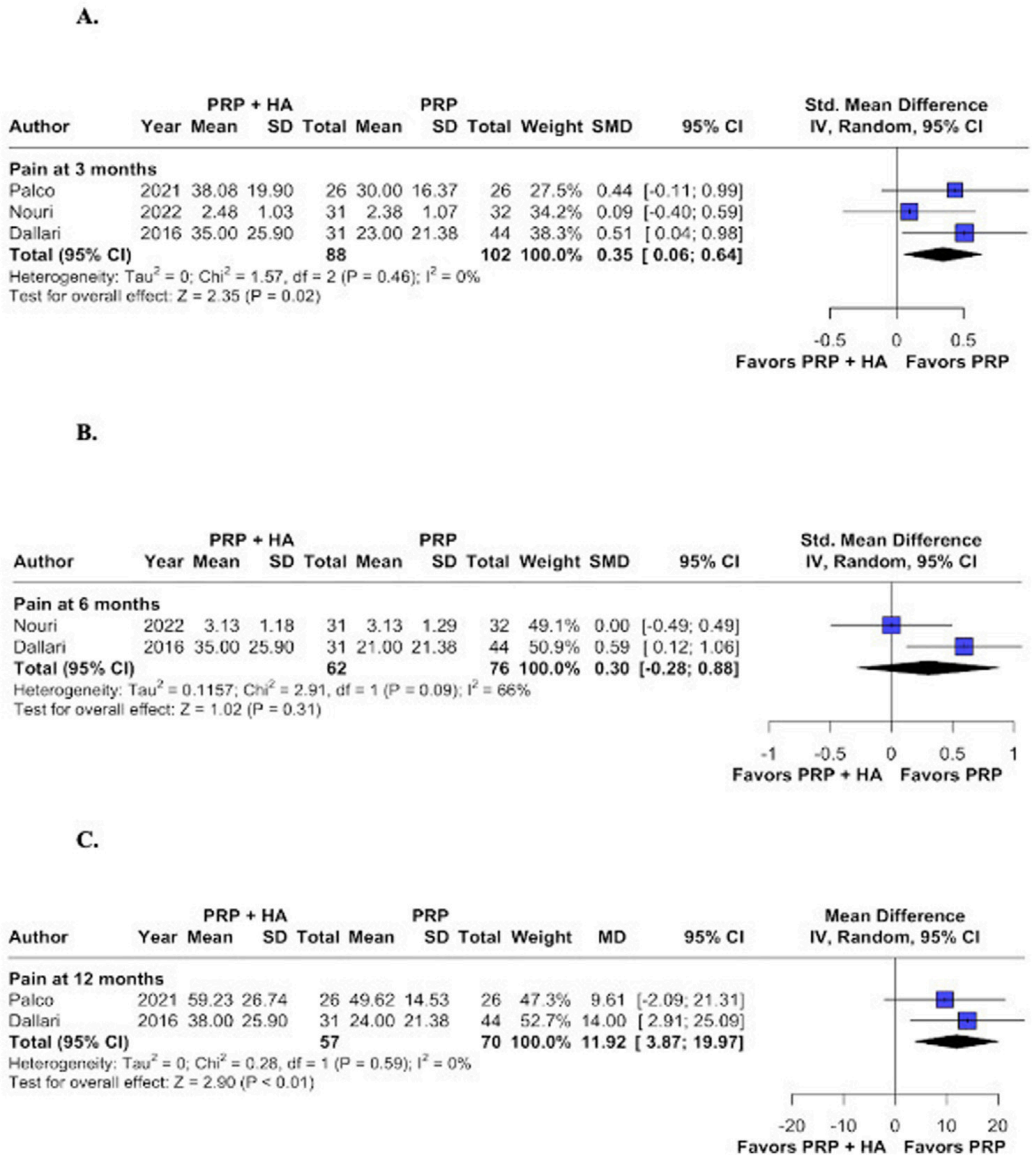
Forest plots for the following endpoints: **(A)** Pain at 3 months; **(B)** Pain at 6 months; **(C)** Pain at 12 months.

Compared with PRP injections alone, combined PRP and HA injections resulted in significantly higher pain at 3 months (MD 0.35; 95% CI: 0.06–0.64; p = 0.02; I^2^ = 0%; Figure 2A) and 12 months (MD 11.92; 95% CI: 3.87–19.97; p < 0.01; I^2^ = 65%; Figure 2B). Nevertheless, the 6-month assessment showed comparable results between the approaches (MD 0.30; 95% CI: −0.28–0.88; p = 0.31; I^2^ = 66%; Figure 2C).

#### 3.2.2 Functional score at 3, 6, and 12 months

**FIGURE 3:**
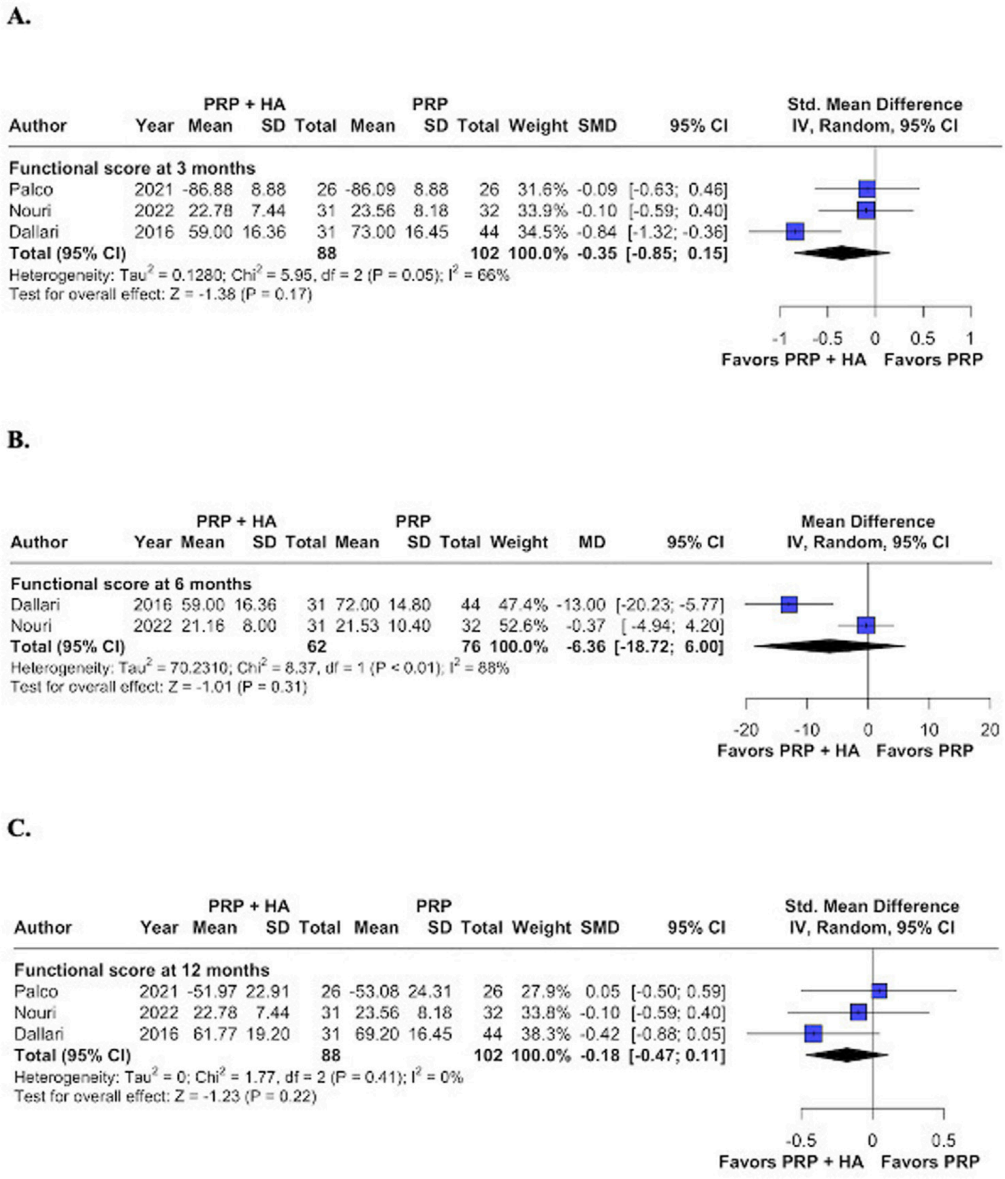
Forest plots for the following endpoints: **(A)** Functional score at 3 months; **(B)** Functional score at 6 months; **(C)** Functional score at 12 months.

The analysis revealed no statistically significant variations in functional outcomes between groups at 3-, 6-, 12- months. The results were as follows: (MD -0.35; 95% CI: −0.85–0.15; p = 0.17; I^2^ = 66%; Figure 3A), (MD -6.36; 95% CI: −18.72–6.00; p = 0.31; I^2^ = 88%; Figure 3B), and (MD -0.27; 95% CI: −0.61–0.07; p = 0.12; I^2^ = 0%; Figure 3C), respectively. All three studies show no improvement in functional scores, likely due to greater joint damage as classified by the Kellgren-Lawrence scale. This damage, particularly the reduced femoro-acetabular joint space, may result in mechanical blockage, limiting the potential impact of the synergistic effect of orthobiological combined treatments. In the study by Dallari et al., patients are distributed across the four K-L grades, while in the other studies, they are distributed across K-L grades 2–3, which justifies a population sample with more advanced joint degeneration (Palco et al., 2021; Dallari et al., 2016; Nouri et al., 2022).

Interpretation of findings about NRS and functional outcomes showed no significant differences, but the confidence intervals are wide, suggesting low statistical power.

### 3.3 Risk of bias evaluation

As shown in Supplementary Figure S1A, the two RCTs exhibited some concerns about this confounding factor. Both were unable to blind the patients given the need to collect blood samples to prepare PRP before its administration. However, given that this was the only point of deviation in the direction of bias - and inherent to the type of intervention - we maintained the level of bias as “some concerns.” Additionally, Supplementary Figure S1B reveals that Palco et al., had some concerns in the ROBINS-I evaluation. These concerns primarily arose from issues related to confounding and participant selection (Sterne et al., 2016; Palco et al., 2021; McGuinness and Higgins, 2021). This figure shows overall concerns, primarily due to confounding issues.

### 3.4 Quality assessment

The GRADE quality assessment revealed very low confidence in pain at 3 months and functional score at 12 months, moderate confidence in both outcomes at 6 months, and high confidence in pain outcome at 12 months. The very low and low confidence ratings were due to serious concerns about inconsistency and imprecision in the studies. Consequently, the actual effects may differ from the estimated effects, as depicted in Supplementary Figure S2. The GRADE assessment was performed using GRADEpro GDT (GRADE pro Guideline Development Tool, McMaster University and Evidence Prime, 2024) (Guyatt et al., 2011).

The GRADE quality assessment indicated very low confidence in the functional score outcome at 3 months, low confidence in both pain at 3 months and functional score at 12 months, moderate confidence in pain and functional score at 6 months, and high confidence in pain at 12 months.

## 4 Discussion

With the inclusion of 3 studies, representing 190 patients, we evaluated the comparative efficacy of intra-articular PRP with versus without HA for managing pain in patients with HOA in any K-L scale awaiting arthroplasty. Our findings revealed: 1) a statistically significant reduction in NRS at 3 and 12 months favoring the PRP group, and 2) no significant improvement in functional scores at any follow-up or in NRS at 6 months. In addition, no significant improvement in pain was noted at 6 months and in functional outcome at any follow-up.

In chronic joint inflammatory processes, there is a reduction in the MW and concentration of HA. When considering viscosupplementation in hip osteoarthritis, studies have shown better clinical response with the use of high molecular weight and severity according to the K-L scale up to 6 months. In this systematic review, we observed that even the use of medium MW (1,500–3,200 kDa) HA was related to a reduction in pain levels for up to 12 months (Schünemann et al., 2013; Wu et al., 2021). However, we observed a lack of standardization in both the molecular weight of HA and the preparation of PRP, which makes a more accurate analysis of the results difficult. A beneficial clinical response is observed when comparing PRP alone with corticosteroids in hip or Knee osteoarthritis in longer follow-ups. In addition to the short duration of pain relief with corticosteroids, it also brings adverse effects that limit their use (Zhao et al., 2020; Freire et al., 2020). However, the use of PRP alone as a regenerative therapy has been more studied, with less than encouraging results, despite knowing its biological properties and safety of use in hip osteoarthritis during a similar period (Pogliacomi et al., 2018).

Regarding knee osteoarthritis of mild and moderate severity, the benefit of dual therapy has been observed in pain control for up to 1 year, while functional improvement is maintained only for the first 3 months (Medina-Porqueres et al., 2021). Given the larger number of studies with bigger sample sizes on the use of orthobiologicals in knee osteoarthritis, we applied this understanding to the hip. It is noted that in knee osteoarthritis, there are better results in pain control and joint function with the addition of HA to PRP than with PRP alone, up to 1 year of administration follow-up. However, combined therapy proved to be superior in long-term follow-up at 2 years. Otherwise, the Gao et al. study shows superiority of the therapy compared to monotherapy, HA, or PRP alone. Still considering this segment, conflicting results have been reported with PRP monotherapy vs. PRP plus HA because there is no standardized procedure for preparation and specific dosage of PRP (Gao et al., 2024; Lana et al., 2016; Howlader et al., 2023; Cole et al., 2017).

Nevertheless, although the basic scientific rationale for adding HA is convincing due to its rheological properties, randomized clinical studies have shown no statistical significance in knee osteoarthritis. It confirmed the benefit in the clinical parameters of pain and function and the improvement both with the isolated use of PRP and with dual therapy using HA of different MW. Even with the comparison between groups, there was no superiority of any intervention (Garcia et al., 2020; Gormeli et al., 2017). The non-discrimination of MW in one of the studies included in our systematic review did not allow the analysis of the results according to this variable since HA with a MW < 3,200 kDa was used. But even so, we observed that regardless of the molecular weight of HA, there was no contribution to pain improvement when associated with PRP. This differs from other studies, corroborating the positive effect of high MW infiltrations in hip joint osteoarthritis (Safali et al., 2024).

This study is the first systematic evaluation of the clinical response of dual therapy (PRP + HA) versus PRP alone for HOA. Considering the paucity of studies in HOA, our results regarding pain outcomes in monotherapy with PRP are similar to the Baria et al. (2022), which saw similar results in knee osteoarthritis (Baria et al., 2022). However, no significant improvement in functional score was found in dual therapy at any follow-up when compared with the PRP group (Filardo et al., 2021). When analyzing the clinical results in younger patients and those with less structural cartilage damage, a better response is seen with the use of PRP alone, but even so there are few studies that support this statement in both knee and hip osteoarthritis. In this way, the results of the functional score using orthobiologics in HOA demonstrate a reduction in pain about specific domains, but considering the final score, no improvement was found (Jacob et al., 2017). However, it is important to notice that this outcome involves many variables, especially within the biopsychosocial context, which may influence these findings (Bennell et al., 2017). While the response to PRP monotherapy in HOA is beneficial for up to 1 year, there is no significant improvement when compared to HA in the same period, when considering intra-articular hip pathologies and any MW of HA (Zaffagnini et al., 2022).

In regulatory and ethical considerations on the use of combined therapy, we have seen that PRP is considered a biological product and may be subject to different regulatory frameworks depending on the country and must be prepared from fresh blood and used during the same medical session and in the same room. HA, on the other hand, is often classified as a medical device or a drug. Given the lack of specific guidelines, it is advisable to consult local regulatory authorities before mixing PRP with HA *ex vivo*. Ensuring compliance with general standards for autologous product preparation and medical device use is crucial. Transparency with patients regarding risks and benefits were essential in all studies. There are ethical concerns and the patients must be appropriately consented in each study.

This study has limitations. The number of published trials available on databases was a significant impediment in formulating this systematic review, with many of the included studies of low confidence ratings not in accordance with the Consolidated Standards of Reporting of Trials (CONSORT) (The CONSORT Statement, 2022), which may limit the reliability of the conclusions. As one of the studies was a retrospective cohort with a reduced sample size, it also represents a factor that prevents a more accurate inference from the general population. Second, the relatively small sample size of 102 patients in the PRP group and 88 patients in the PRP + HA group in the final analysis of the selected studies could affect the generalizability of the results. Third, the variability in study design (including two RCTs and one cohort study) and follow-up duration may introduce methodological and statistical heterogeneity. Considering the different PRP preparation techniques, the MW of HA and the frequency of joint infiltrations that show variation in results also contribute to high heterogeneity. Fourth, the scales used to analyze pain and functional deficit of the hip differed across studies, and this is one more factor that introduces bias in the outcomes. The lack of significant improvement in functional scores suggests that while pain management might be enhanced, the overall clinical benefit remains unclear. These limitations can be addressed by using orthobiologicals with standardized PRP preparation methods and higher molecular weight HA in patients with similar degrees of hip osteoarthritis. This approach could improve the measurement of outcomes, aiming to enhance clinical applications and provide greater benefits to patients.

## 5 Conclusion

The outcomes analyzed in this meta-analysis suggest that the utilization of dual therapy versus PRP in the context of HOA confers a significant reduction in pain. However, this does not apply to the improvement in quality of life-related to the application of functional questionnaires at any follow-up time without statistical significance. The addition of HA did not influence the improvement in clinical response to pain and function outcomes. Finally, we found a significant reduction in pain at 3- and 12- months, favoring joint infiltration with PRP alone, relative to HA plus PRP, which leads to maintenance of clinical improvement for a longer period, even considering it is a chronic degenerative disease. Although our results showed promising results favoring the use of orthobiologics, we understand that the low number of trials and sample size limits make our conclusions preliminary warranting more trials with larger population samples. Nevertheless, large sample sizes are also warranted to validate our findings about the benefit of HA plus PRP in HOA before these results are implemented in clinical practice. We recognize that standardized PRP/HA protocols are needed before strong clinical recommendations can be made that provide a more balanced interpretation. Therefore, high-quality RCTs and longer follow-up periods (>12 months). Are warranted to understand the clinical benefit of HA plus PRP in HOA.

## Data Availability

The original contributions presented in the study are included in the article/Supplementary Material, further inquiries can be directed to the corresponding author.
