# Supplementary material for: Platelet-rich plasma with versus without hyaluronic acid for hip osteoarthritis: a systematic review and meta-analysis
Source: Front Bioeng Biotechnol. 2025 Mar 24;13:1545431. doi: 10.3389/fbioe.2025.1545431 (PMC11980421; doi:10.3389/fbioe.2025.1545431)
Supplement: Supplementary file 1 [file Table1.docx]

**Supplementary Table 1**. Search strategies for each database.

| **Database** | **Search strategy** |
| --- | --- |
| Pubmed | ("hip osteoarthritis" OR osteoarthritis) AND (platelet OR PRP) AND ("hyaluronic acid" OR viscosupplementation) |
| Embase | ("hip osteoarthritis" OR osteoarthritis) AND (platelet OR PRP) AND ("hyaluronic acid" OR viscosupplementation) |
| Cochrane | ("hip osteoarthritis" OR osteoarthritis) AND (platelet OR PRP) AND ("hyaluronic acid" OR viscosupplementation) |
